# Assessing temporal variability and controlling factors of the sediment budget of a small agricultural catchment in Northern France (the Pommeroye)

**DOI:** 10.1016/j.heliyon.2019.e01407

**Published:** 2019-03-26

**Authors:** Edouard Patault, Claire Alary, Christine Franke, Arnaud Gauthier, Nor-Edine Abriak

**Affiliations:** a IMT Lille Douai, Univ. Lille, EA 4515 – LGCgE – Civil Engineering and Environmental Department, F-59000 Lille, France; b MINES ParisTech, PSL Research University, Center of Geosciences, 35 rue Saint-Honoré, 77305 Fontainebleau Cedex, France; c Normandie Univ, Rouen, UNIROUEN, UNICAEN, CNRS, M2C, FED-SCALE, Rouen, France; d Département des Sciences de la Terre, Université de Lille 1, Villeneuve d’Ascq, France

**Keywords:** Environmental science, Agriculture, Earth science, Geoscience, Hydrology

## Abstract

A high-frequency monitoring station was implemented at the outlet of the small catchment of the Pommeroye (0.54 km^2^) in Northern France to study erosion by runoff and hydro-sedimentological responses to heavy rainfall events in the context of Quaternary loess deposits. The aim of this experimental work is to assess the temporal variability of sediment yield and to identify the factors controlling the hydro-sedimentary response. To achieve this goal, statistical and hydro-sedimentary dynamic analyses were performed. During two years of monitoring (April 2016–April 2018), 48 flood events were recorded. The specific sediment yield (SSY) is highly variable and was evaluated to 29.4–70 t km^-2^ yr^−1^ which is conventional for the study region. Most of the sediment yield was produced in winter (55%) and autumn (42%). Only 3% of SSY were produced during spring and summer periods. According to our results, only 6% of the erosive events are responsible for the transport of more than 40% of the sediment flux recorded at the outlet. This underlines the temporal variability of the hydro-sedimentary production in small agricultural catchments for which most of the hydro-sedimentary flux is produced during a limited number of events. The results of statistical analyses show that the total amount of rainfall and the duration of a rainfall episode are the main controlling factors on the hydro-sedimentary response. Our results also suggest that the rainfall kinetic energy better reflects the sediment detachment, and that the 48 h-antecedent rainfall is not linked to the hydro-sedimentary response.

## 1 Introduction

In the North-Western European loess plateau and in particular in the North of the Paris Basin, erosion of agricultural land is a serious environmental issue (Evrard et al., 2007; Delmas et al., 2012). Every year, numerous natural disasters occur due to various erosion processes which result in on-site and off-site problems including the loss of fertile soils, the silting of riverbeds and dams, as well as infrastructure and property damage by muddy floods (Capra, 1993). The most common type of soil erosion pattern observed in these territories are (i) sheet erosion, (ii) rills, (iii) interrills, (iv) classical gullies, and (v) ephemeral gullies (Foster, 1986). So far, the focus was drawn to rill/interrill erosion, but recently a growing interest has focused on ephemeral gullies (Poesen, 1993; Nachtergaele et al., 2002; Valentin et al., 2005; Castillo and Gomez, 2016), since they have been recognized as a major contributor to sediment yield in small agricultural catchments in the European loess belt (Poesen et al., 1996). Classical survey methods to evaluate sediment production on small catchments are aerial photography (Nachtergaele and Poesen, 1999; Marzolff et al., 2011), terrestrial photography (Frankl et al., 2011), terrestrial laser scanning (Li et al., 2017), airborne laser scanning (Goodwin et al., 2017), or direct measurements of channel volumes (Valcárcel et al., 2003). However, these methodologies are not suitable for quantifying the sediment yield at the catchment scale (Vandaele and Poesen, 1995). Understanding and quantifying the dynamics of suspended sediment transport is essential for controlling soil erosion and implementing effective mitigation practices to reduce stream suspended matter and associated pollutants discharge. Currently, catchment monitoring was successfully used by several authors (Nadal-Romero et al., 2008; Estrany et al., 2009; Nu-Fang et al., 2011; Giménez et al., 2012; Sherriff et al., 2015) to quantify erosion processes in agricultural catchments and to assess the relationships between hydro-sedimentological parameters. These studies have reported on sediment transfer that show high temporal and spatial variabilities, and the fact that only few erosive events are responsible for the majority of the sediment export (Gay et al., 2014; Bagarello et al., 2017; Grangeon et al., 2017). They also report on complex correlations between rainfall characteristics and the hydro-sedimentary response. Some attempts have been made to evaluate the variability of the hydro-sedimentary response at larger scale in the context of agricultural plains of North-Western Europe (Cerdan et al., 2010; Vanmaercke et al., 2011), but according to Poesen (2018), very few studies have measured the water erosion dynamic on relatively small catchments (0.01–1000ha). At this scale range, there is a shift in the dominance of particular erosion processes. As reported by Vanmaercke et al. (2011), small headwater lowland catchments may exhibit significant sediment export. According to Grangeon et al. (2017), the spatial and temporal dynamics of sediment fluxes must be further studied to improve our understanding of the possible connection/disconnection between the water and the sediment transport pathways, in particular for areas where intensive agriculture is predominant and runoff can be generated by soil saturation (Gay et al., 2016). Some recent studies already point out that connectivity between sediment sources and rivers is essential for soil redistribution (Landemaine et al., 2015; Foucher et al., 2015; Heckmann et al., 2018). Sediment flux data for such small catchments are therefore essential for better understanding the linkages between soil erosion processes and suspended sediment transport of larger rivers (Verstraeten and Poesen, 2001). However, quantification of the sediment yield at this scale might be challenging, especially if there is a lack of a perennial hydrographic network in the studied catchment. Generally, the characterization and quantification of sedimentary flow is limited to experimental data of spot samples at the outlet of the watershed and therefore does not necessarily take into account the internal dynamics of the basin (Foucher, 2015).

In the context of the Canche River watershed (Northern France), erosion of agricultural lands may lead to particularly devastating muddy flows that generate an important export of sediments and a high material costs for the local communities. Since 1983, 1100 municipalities were stroke by heavy muddy flows. So far, any systematic study was conducted to better understand the genesis of these phenomena. Thus, the local Water Agency expressed the clear need for high-resolution data on the Canche River watershed, to be able to quantify the sediment exported by mudflows, to define their respective temporal variability, and to identify the main controlling factors.

Nevertheless, the understanding of the hydro-sedimentary fluxes variability and the associated factors on the Canche River watershed first requires the monitoring and understanding of processes at the scale of the experimental catchment. The goal of this study was to deploy a high-frequency monitoring station in a challenging context since the small studied catchment (0.54 km^2^) is lacking a perennial hydrographic network. The hydro-sedimentary behavior was characterized with a high-temporal resolution (6 min). To this end, runoff events are monitored over two hydrological years (April 2016 to April 2018). This allows to: (i) quantify short-term changes between different runoff events, (ii) identify the main controlling factors, and (iii) determine the temporal variability. These results are crucial to be able to better address the existing soil erosion problems in northern France related to rain events runoff.

## 2 Study area

**Fig. 1:**
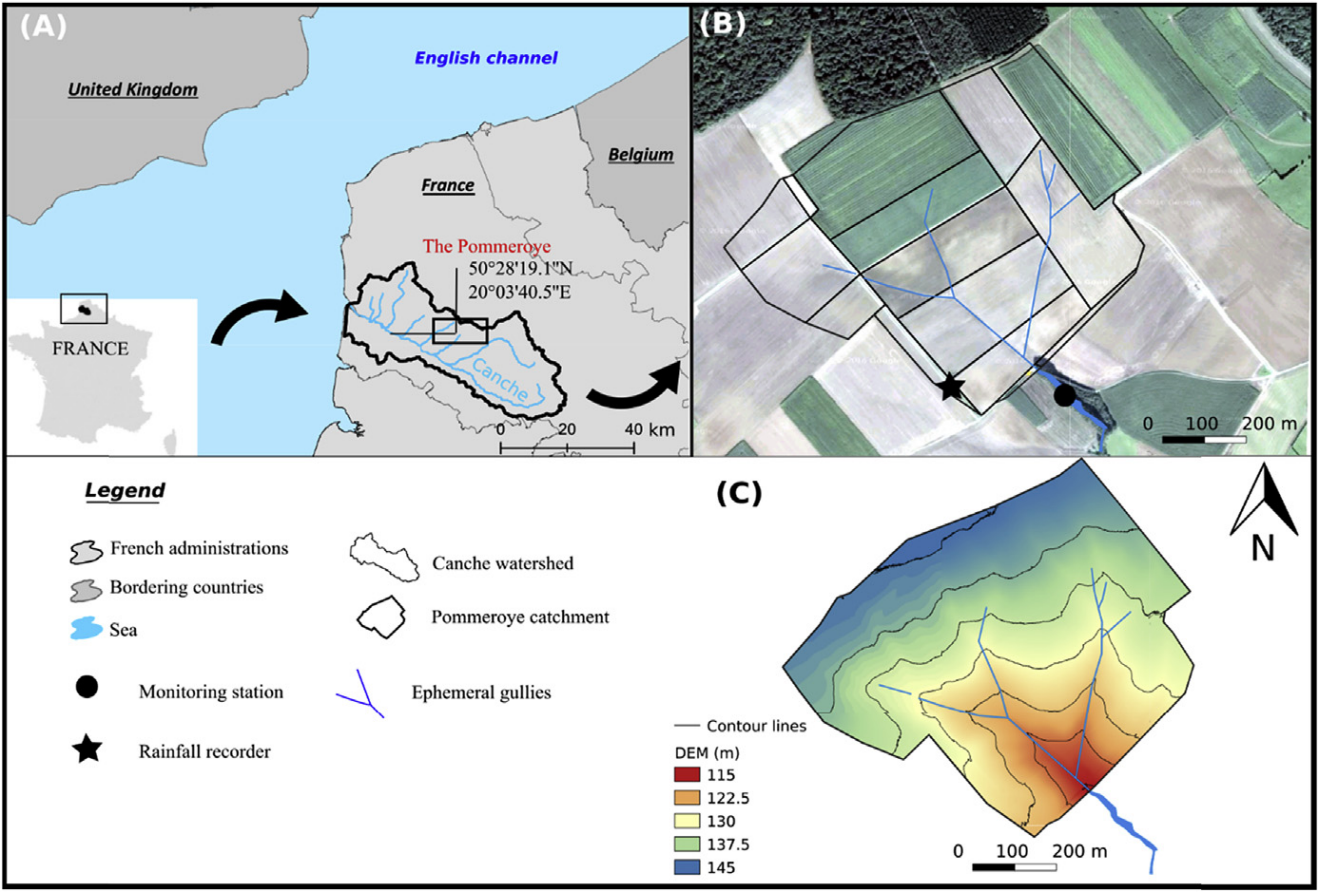
(A) Overview on the study region, (B) map of the Pommeroye catchment showing the location of the ephemeral gullies and instruments used in this study, (C) Digital Elevation Model (DEM) of the study site (cell size: 50 cm).

The Pommeroye catchment (0.54 km^2^) is situated in the European loess belt in Northern France (Fig. 1A). It is a sub-catchment of the Canche River watershed (1274 km^2^). The dominant climate is oceanic and the average annual temperature in this region is 11 °C. The thermal amplitude is low, with soft winters and cool summers. The annual rainfall amount is 1000 ± 150 mm yr^-1^. An ephemeral gully network is well-established and recurrent, resulting from the junction of rills that form a dendritic channel pattern (Fig. 1B). The elevation of the study area ranges from 115 to 145 m and the average slope is 4.2% (Fig. 1C). The soil is constituted of Pleistocene silt which overlays the chalky soil of the Seno-Turonian (Beckelynck, 1981). Grain size analyzes carried out by Patault (2018), on 22 agricultural soil samples, in the Canche watershed, show that agricultural soils are composed of clay (5%), silts (54%), and fine sands (41%). The medium-textured soils (loamy soils) tend to be most erodible because they have high amounts of silts and very fine sand. These soils tend to have the highest soil erodibility factor in Europe (K > 0.055 t ha h ha^−1^ MJ^−1^ mm^−1^; Panagos et al., 2014). The study site is exclusively occupied by arable land, divided into 14 fields. The dominant crops here are cereals, winter and spring barley, rape seed, and mustard.

## 3 Material & methods

### 3.1 The monitoring station

A monitoring station consisting of an exponential Venturi channel and an approach channel has been installed on March 31, 2016 at the outlet of the Pommeroye catchment to record the flow discharge and suspended sediment concentration (SSC). The hypothetic maximum flow discharge was estimated using the rational equation for peak discharge (Thompson, 2006). The rational method is a simple technique for estimating a design discharge from a small catchment. The method is based on the simple equation that relates the runoff-producing potential of the catchment, the average intensity of rainfall for a particular length of time (the time of concentration), and the catchment area (Eq.1):

(1) Q=C×i×A

Where Q is the design discharge (m^3^ s^−1^), C is the runoff coefficient (between 0 and 1; a value of 0.1 is usually applied for agricultural lands), i is the design rainfall intensity (mm h^−1^), and A is the catchment drainage area (ha). The design rainfall intensity is defined using the Montana coefficients (Eq.2):

(2) $$i=a\times t^{-b}$$

Where t is the concentration time (t = 0.34 h), a and b are the Montana coefficients at the closest meteorological station. Here, the closest station for which the coefficient are available was the station of Le Touquet-Paris-Plage (a = 23.3 and b = 0.67). Using the rational method, the design discharge was evaluated to 0.72 m^3^ s^-1^, which is slightly over-evaluated considering that the station of Le Touquet-Paris-Plage is located in an area that is affected by slightly higher rainfall than the study area. The Venturi channel VII provided by *ISMA* allowed a maximum discharge of 0.40 m^3^ s^-1^, which was considered in adequacy with the objectives of our study.

**Fig. 2:**
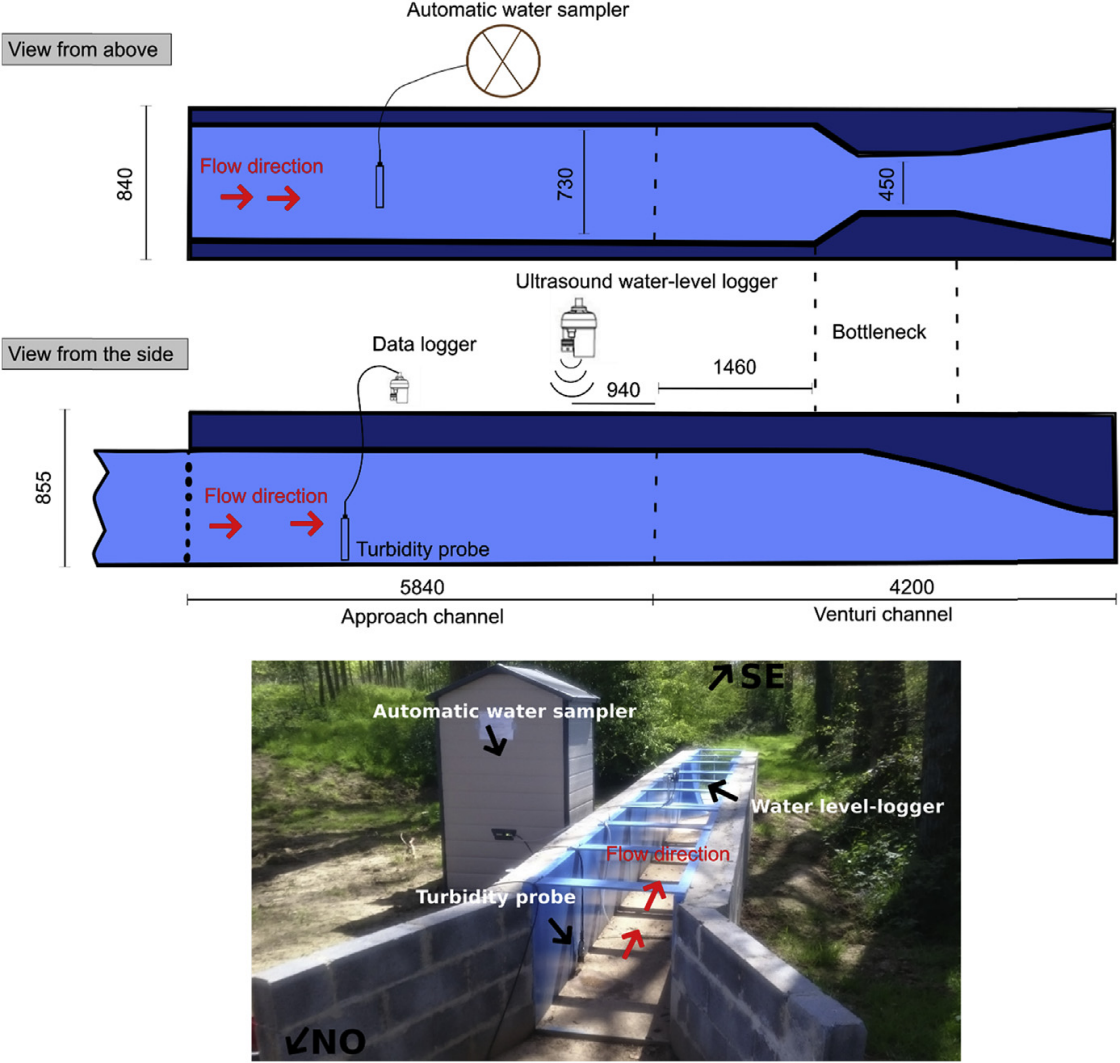
Schema and picture of the monitoring station at the outlet of the Pommeroye catchment viewed from different perspectives (the dimensions are given in mm).

According to the calculated maximum flow discharge, a Venturi channel (*Venturi channel ISMA VII*) of suitable dimensions for measuring the hypothetic discharge has been selected (Fig. 2). The length of the approach channel was established considering the *ISO 4359 standard* (AFNOR, 1986) that requires an approach length of at least 5 times the width of the channel approach, upstream of the load measurement zone. This corresponds to three to four times the maximum height to be measured, upstream of the Venturi's contraction. The approach channel assures the passage of the torrential flow generated by the various constraints of ground, to a river regime at the entrance of the Venturi channel, necessary for the validity of an adapted monitoring. The Venturi and the approach channel are positioned horizontally, without any slope, in the longitudinal and transversal direction. They are perfectly aligned and are thus not affected by any profile changes. An ultrasound water-level logger (*Ijinus LNU 300-X*) records the water height in the Venturi channel.

Runoff discharge is calculated for each time step in the Venturi channel using the following equation (Eq.3) and a conversion table (Table 1) provided by the manufacturer (*ISMA*; *www.isma.fr*) of the Venturi channel. The relation between height and flow discharge was defined by the manufacturer and validated using in-situ experimentation and 3-D modelling (Dufresne et al., 2010):

(3) $$Q=\;-7,223\times h+2873,2\times h^{2}-766\times h^{3}+770\times h^{4}$$

Where Q is the flow discharge in the Venturi channel (m^3^ h^−1^) and h the water height (m).

**Table 1:** Conversion table of height (mm) and flow discharge (m^3^ h^−1^) for the Venturi channel *ISMA VII*, provided by the manufacturer.

h (mm)	Q (m^3^ h^−1^)	h (mm)	Q (m^3^ h^−1^)
73	14.51	410	448.99
80	17.45	425	482.22
95	24.65	440	516.68
110	33.06	455	552.39
125	42.68	470	589.34
140	53.50	485	627.56
155	65.50	500	667.06
170	78.69	515	707.86
185	93.05	530	749.97
200	108.59	545	793.41
215	125.29	560	838.19
230	143.17	575	884.35
245	162.20	590	931.88
260	182.41	605	980.83
275	203.77	620	1031.20
290	226.31	635	1083.02
305	250.01	650	1136.32
320	274.88	665	1191.12
335	300.92	680	1247.44
350	328.15	695	1305.31
365	356.56	710	1364.76
380	386.17	725	1425.82
395	416.97	732	1454.87

The range of the ultrasound water-level is 0–792 mm in the channel and the resolution is 2 mm. The SSC is estimated using a turbidity probe (*Odeon/Aqualabo*); its accuracy is lower than 5% NTU. Regular cleaning of the probe head is carried out after each flood event. An automatic water sampler (*ISCO 3700*; 24 × 1 L) is used to collect water samples during flood events. The sampler is coupled to the water-level sensor and only operates when water flows through the Venturi channel. All sensors are connected to a data logger (*Ijinus Log0500*). The measured parameters are recorded every 6 min. Information is downloaded every two weeks on a laptop computer (*Software Avelour 6.0.4*). A tipping-bucket rain gauge (*Précis mécanique; model: 303x*; resolution: 0.2 mm) was also installed in the catchment close to the station (see star symbol in Fig. 1B).

**Fig. 3:**
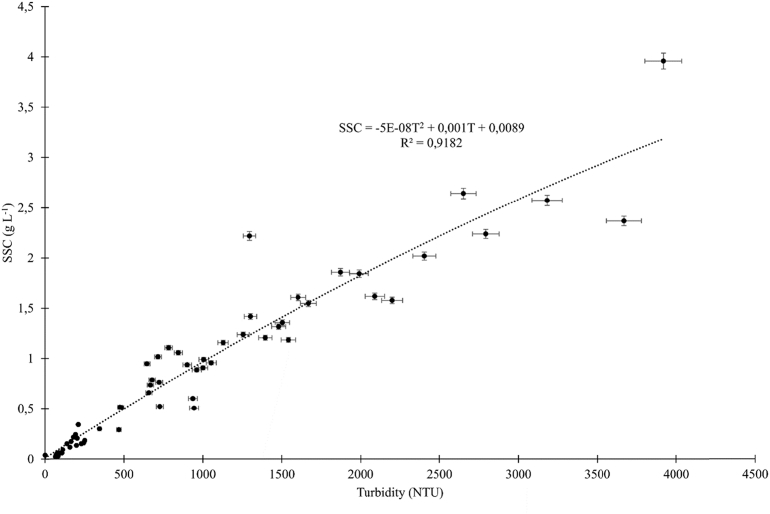
Correlation curve between turbidity (NTU) and suspended sediment concentration (g L^−1^) for the water samples sampled in the Venturi channel.

To estimate the SSC over time, 61 water samples were sampled using the automatic water sample. 100 mL of water are filtered in the laboratory using dehydrated cellulose nitrate filters, previously dried in an oven (70 °C during 48 h; pore size 0.45 μm). Subsequently, the filters were dried again (70 °C during 48 h) and weighed. The weight difference allows evaluating the concentration of the material in the sample. A correlation curve is defined between T the turbidity data (NTU) measured in the field and SSC corresponding to the suspended sediment concentration (g L^−1^) obtained in the laboratory (Fig. 3).

Suspended sediment flux (SSF) is then calculated using Eq. (4):

(4) $$SSF=\;Q\times SSC\times 10^{3}$$

With Q the flow discharge in m^3^ s^−1^ and SSF the instant suspended sediment flux in g s^−1^. The sediment yield (SY; in g) is evaluated for each event as (Eq. 5):

(5) $$SY=\;\overset{t_{f}}{\underset{t_{0}}{\int }}SSF\;dt$$

With $t_{0}$ and $t_{f}$ corresponding to the beginning and the end of the event considered. Then, the SY is converted in kg to facilitate the comparison.

### 3.2 Analysis of variables

During the two years of monitoring (April 2016–April 2018), a total of 48 runoff events were recorded. For each event, multiple variables were extracted, related to: (i) duration rainfall event (R_time_, min), (ii) rainfall amount (Ra, mm), (iii) maximum 6 min rainfall intensity (Ri_max_, mm h^−1^), (iv) rainfall amount 48 h before the beginning of the investigated event (Ra_48_, mm), (v) mean flow and peak flow (Q_mean_, Q_max_, m^3^ h^−1^), (vi) mean and maximum SSC concentration (SSC_mean_, SSC_max_, g L^−1^), (viii) sediment yield (SY, kg), and (ix) total runoff (R_tot_, m^3^). According to Morgan and Duzant (2008) rainfall detachment is better reflected by rainfall kinetic energy (RKE, J m^−2^ mm^−1^) than by rainfall intensity, thus the RKE was derived from the Ri_max_ value using the equation of Brandt (1989; Eq. 6), that was tested in the similar environmental context of the Loire River watershed in France (Grangeon et al., 2017):

(6) $$RKE=8.95+8.44\times \log_{10}Ri_{max}$$

The rainfall erosivity index ($EI_{30})$ was also considered as it measures both rainfall's kinetic energy and intensity to describe the effect of rainfall on sheet and rill erosion (Wischemeir and Smith, 1978). It is a product of kinetic energy of a rainfall event (E) and its maximum 30-min intensity (I_30_). The rainfall erosivity index (EI_30_; MJ mm ha^−1^ h^−1^) was calculated for each event using Eq. (7) given by Brown and Foster (1987):

(7) $$EI_{30}=\left(\overset{m}{\underset{r=1}{\sum }}e_{r}\times v_{r}\right)\times I_{30}$$

Where $e_{r}$ is the unit rainfall energy (MJ ha^−1^ mm^−1^) and $v_{r}$ is the rainfall volume (mm) during the r-th period, divided into m parts. $I_{30}$ is the maximum rainfall intensity during a 30-min period of the rainfall event (mm h^−1^). $I_{30}$ is evaluated using the high-resolution rainfall data (6 min) which provides better $EI_{30}$ values according to Panagos et al., 2015a, Panagos et al., 2015b, Panagos et al., 2015c.

The unit rainfall energy $e_{r}$ is calculated for each time interval as follows (Brown and Foster, 1987):

(8) $$e_{r}=0.29\times [1-(0.72\times e^{-0.05\times i_{r}})]$$

where i is the rainfall intensity during the time interval (mm h^−1^).

### 3.3 Pearson correlation matrix and principal component analysis (PCA)

Statistical analyses were performed using the statistical software *R*^1^ and the following packages: *FactoMiner*^2^ and *Corrplot*^3^. Pearson correlation matrix is used to evaluate the linear dependency between multiple variables simultaneously. The result is given using the Pearson correlation coefficient r which reflects the linear correlation between two variables. The coefficient is calculated using the covariance of two variables divided by the product of their standard deviations (Eq. 9):

(9) $$r=\frac{\sum_{i=1}^{n}\left(x_{i}-\bar{x}\right)\left(y_{i}-\bar{y}\right)}{\sqrt{\sum_{i=1}^{n}\left(x_{i}-\bar{x}\right)²}\sqrt{\sum_{i=1}^{n}\left(y_{i}-\bar{y}\right)²}}$$

Where n is the sample size; $x_{i}$ and $y_{i}$ are the values of the sample; $\bar{x}$ and $\bar{y}$ are the mean values of the sample. A value of 1 implies that a linear equation describes the perfect relationship between $x_{i}$ and $y_{i}$, with all data points lying on a line for which $y_{i}$ increases as $x_{i}$ increases. A value of −1 implies that all data points lie on a line for which $y_{i}$ decreases as $x_{i}$ increases. A value of 0 implies that there is no linear correlation between the variables.

### 3.4 General characteristics of runoff events

#### 3.4.1 First year of monitoring

Between April 2016 and April 2017, 22 runoff events were recorded: seven occurred in spring, three in summer, four in autumn, and eight in winter. The main characteristics of these events are summarized in Table 2 and are described in detail in the following text:

i .The duration of runoff events ranged from 126 to 7200 min in total with a median value of 534 min. Five events (22%) showed a duration longer than 1000 min, and five events (22%) correspond to shorter period (less than 360 min), one event exceeded 2000 min.
ii .The rainfall amount ranged from 6 to 103.8 mm with a median value of 16.1. Eight events (36%) exceeded 20 mm, and five events (22%) had values lower than 10 mm.
iii .The maximum 6 min rainfall intensity ranged from 3 to 76 mm h^-1^, and three events (13%) exceeded 20 mm h^-1^. The amount of precipitation 48 h before the beginning of an event ranged from 0.2 to 25.4 mm. Seven events (32%) showed a rainfall amount 48 h before the beginning of an event that was higher than 10 mm.
iv .The peak flow ranged from 0.6 to 378.3 m^3^ h^-1^ and the mean flow ranged from 0.11 to 94.5 m^3^ h^-1^. Ten events (45.5%) showed values of a peak flow exceeding 100 m^3^ h^-1^.
v. The maximum SSC ranged from 0.15 to 5 g L^-1^ and the mean SSC ranged from 0.06 to 1.6 g L^-1^. Eleven events (50%) showed values of maximum SSC exceeding 3 g L^-1^.
vi. The sediment yield values ranged from 0.07 to 7131.9 kg and were extremely variable. Three events (13.6%) exceeded 1500 kg and one event exceeded 2500 kg. This event represents 44.8% of the total sediment discharge for the first year of monitoring.
vii. The total runoff ranged from 0.57 to 8630.5 m^3^. Four events (18.2%) exceeded 1000 m^3^ and one event exceeded 2500 m^3^.
viii. The rainfall kinetic energy ranged from 13 to 24.8 J m^-2^ mm^−1^. Events with a high RKE (>20) are mainly observed during the spring season, and do not correspond to the events with the highest sediment yield recorded at the outlet.
ix. The rainfall erosivity index (EI_30_) ranged from 0.7 to 236.2 MJ mm ha^−1^ hr^−1^ with a mean value of 25.3 MJ mm ha^−1^ hr^−1^. Highest value of EI_30_ is observed in May (236.2 MJ mm ha^−1^ hr^−1^) during an intensive rainfall event where Ri_max_ reached 76 mm h^-1^. The sum of EI_30_ during the first year of monitoring round up to 557.45 MJ mm ha^−1^ hr^−1^.

**Table 2:** Main characteristics of the 22 flood events recorded in the Pommeroye catchment between April 2016 and April 2017.

Date	R_time_ (min)	Ra (mm)	Ri_max_ (mm h^−1^)	Ra_48_ (mm)	Q_mean_ (m^3^ h^−1^)	Q_max_ (m^3^ h^−1^)	SSC_mean_ (g L^−1^)	SSC_max_ (g L^−1^)	SY (kg)	R_tot_ (m^3^)	RKE (J m^−2^ mm^−1^)	EI_30_ (MJ mm ha^−1^ hr^−1^)
12-Apr-16	192	8.1	9	5.8	15	42	1.2	4.1	71.2	31.4	17	4.3
15-Apr-16	366	13.8	12	0.6	5.9	10.8	0.4	2.1	5.8	8.2	18.1	6.5
11-May-16	288	32.6	76	7.2	22.2	150.8	1.6	5	652.7	186.5	24.8	236.2
22-May-16	204	9.8	6	2.8	0.25	0.6	0.06	0.15	0.07	0.8	15.5	3.5
31-May-16	1110	30.6	14	0.2	22.6	147	0.7	3	235.8	106.2	18.6	9.7
3-Jun-16	126	6	12	1.4	1.91	6.24	0.13	0.8	1.4	4.6	18.1	4.8
20-Jun-16	372	8.7	3	3.4	3.7	9.14	0.1	0.27	4.3	23.3	13	5.2
23-Jun-16	245	12.9	21	5.3	13.6	60.7	0.5	2.69	80.2	69.3	20.1	42
2-Aug-16	1968	68.6	10	18.4	12.4	108.6	0.32	4.58	590.9	407.5	17.4	39.1
9-Sep-16	324	27	24	0.2	4.5	6.2	0.5	2.7	6.8	12.2	20.6	50.8
20-Oct-16	390	16.6	4	11.4	3.08	12.9	1.06	4.03	30.4	20.3	14	3.9
7-Nov-16	1434	17.5	8	0.9	0.11	0.99	0.5	3.02	0.6	0.57	16.6	8.2
16-Nov-16	540	9.7	3	1.2	0.34	2.54	0.38	1.63	1.86	3.7	13	3.8
17-Nov-16	7200	103.8	18	10.9	75.2	378.3	0.41	4.02	7131.9	8630.5	19.5	83.5
12-Jan-17	528	22	12	2	55.18	190.9	1.35	4.9	1597.6	772.6	18.1	16.1
4-Feb-17	432	6.8	6	11.8	1.96	13.7	0.12	0.89	8.99	16.2	15.5	1.32
5-Feb-17	702	25.2	6	11.8	94.5	235.8	0.74	3.7	2119.7	1767.4	15.5	8.43
7-Feb-17	804	10.6	8	25.4	34.4	209.8	0.37	3.4	627.3	561.3	16.6	4.8
27-Feb-17	852	15.6	8	1	3.9	37.5	0.42	3.7	87.9	57.9	16.6	0.7
28-Feb-17	1002	21.6	8	17	68.4	221.8	0.35	1.59	991.3	1957.1	16.6	9.4
5-Mar-17	1050	16.8	10	11.4	36.1	227.9	0.35	3.82	1059.5	1274.6	17.3	10.5
8-Mar-17	894	14.6	4	0.6	50.8	189.5	0.19	1.27	595.1	2031.8	14	4.7
Mean	955.6	22.7	12.8	6.9	23.9	102.9	0.54	2.8	722.8	815.6	17.1	25.3
Std dev.	1467.2	22.6	15.2	7	28	108.4	0.42	1.5	1544.9	1873.3	2.7	51.4

#### 3.4.2 Second year of monitoring

Between April 2017 and April 2018, 26 runoff events were recorded: twelve events occurred in autumn and fourteen in winter. The main characteristics of these events are summarized in Table 3 and are described in detail in the following text:

i. The duration of runoff events ranged from 72 to 4098 min in total with a median value of 522 min. Eight events (30%) showed a duration longer than 1000 min, and seven events (27%) correspond to shorter flooding (less than 360 min).
ii. The rainfall amount ranged from 2 to 55.2 mm with a median value of 12.2 mm. Eight events (30%) show values over 20 mm, and eleven events (42%) were lower than 10 mm.
iii. The maximum 6 min rainfall intensity ranged from 1 to 32 mm h^-1^ and five events exceeded 20 mm h^-1^ 48 h-antecedent rainfalls ranged from 0 to 31.8 mm and twelve events (46%) showed 48 h-antecedent rainfalls higher than 10 mm.
iv. The peak flow ranged from 60.4 to 348.6 m^3^ h^-1^ and the mean flow ranged from 14.7 to 110.1 m^3^ h^-1^ 24 events (92%) showed a value of peak flow exceeding 100 m^3^ h^-1^.
v. The maximum SSC range from 0.5 to 5 g L^-1^ and the mean SSC ranged from 0.08 to 1.31 g L^-1^. Twelve events (46%) showed values of maximum SSC exceeding 3 g L^-1^.
vi. The sediment yield values ranged from 54.8 to 7577.5 kg. Nine events (34%) exceeded 1500 kg and three main events (11%) exceeded 3000 kg.
vii. The total runoff ranged from 141 to 5255.5 m^3^. Eleven events exceeded 1000 m^3^ and two main events exceeded 2500 m^3^.
viii. The rainfall kinetic energy ranged from 8.9 to 21.7 J m^-2^ mm^−1^ and show a biggest variability than the previous year. Highest RKE are principally observed between October and December 2017.
ix. The rainfall erosivity index (EI_30_) ranged from 0.9 to 81.8 MJ mm ha^−1^ hr^−1^ with a mean value of 12.5 MJ mm ha^−1^ hr^−1^. The sum of EI_30_ during the first year of monitoring round up to 314.15 MJ mm ha^−1^ hr^−1^, which is considerably lower than the previous year.

**Table 3:** Main characteristics of the 26 flood events recorded in the Pommeroye catchment between April 2017 and April 2018.

Date	R_time_ (min)	Ra (mm)	Ri_max_ (mm h^−1^)	Ra_48_ (mm)	Q_mean_ (m^3^ h^−1^)	Q_max_ (m^3^ h^−1^)	SSC_mean_ (g L^−1^)	SSC_max_ (g L^−1^)	SY (kg)	R_tot_ (m^3^)	RKE (J m^−2^ mm^−1^)	EI_30_ (MJ mm ha^−1^ hr^−1^)
20-Oct-17	600	4.8	6	1.3	16.9	74.6	0.11	1.3	54.8	211	15.5	0.9
22-Oct-17	600	28.9	32	7.8	20	170	0.17	2.2	167.1	210	21.7	11.8
23-Oct-17	966	7.1	1	28.9	14.7	60.4	0.08	0.5	55.7	461	8.9	1.3
14-Nov-17	2292	5.6	6	0.5	17.7	144.8	0.12	1.8	200.5	670	15.5	1.7
18-Nov-17	72	15.2	10	0.8	27.5	183.1	0.24	2.4	137.3	184	17.4	8.7
20-Nov-17	822	23.2	8	19	33.3	140	0.26	2.3	300.9	786	16.6	12
27-Nov-17	2238	54.8	24	0.2	56.5	308.5	0.59	4.1	3529	2234	20.6	81.8
29-Nov-17	1158	14.6	10	6.8	36	153	0.3	5.0	354.8	659	17.4	8.95
7-Dec-17	498	8.8	6	0.6	24.8	177.2	0.22	3.2	120.2	141	15.5	1.4
10-Dec-17	1338	14.4	10	0	46.6	348.6	0.48	4.0	1799	961	17.4	5.4
11-Dec-17	1218	20.4	6	14.4	28	285	0.23	4.2	1064.2	1186	15.5	6.6
13-Dec-17	4098	55.2	8	20.6	64.9	325.9	0.67	4.3	7577.5	5255.5	16.6	25.5
27-Dec-17	372	5.2	4	6	48.1	117.7	0.45	3.4	311.3	442.5	14	0.9
29-Dec-17	420	15	22	3	91.2	321.5	1.03	4.3	2239.8	1177.1	20.3	25.6
30-Dec-17	366	6.2	4	15.4	54.5	172	0.52	2.2	677.7	807	14	1.3
31-Dec-17	2694	37.2	30	21.4	80.8	335.7	0.89	3.8	6919.7	4245.1	21.4	62.6
3-Jan-18	192	10.4	10	17.2	110.1	294.4	1.31	3.1	3084.4	1357.3	17.4	6.9
4-Jan-18	336	21.2	6	13.6	86.9	239.5	0.99	3.2	2353.1	1286.9	15.5	7.8
5-Jan-18	360	8	20	31.8	73.1	328.3	0.79	4.3	1886.5	1104.8	19.9	13
6-Jan-18	330	7.4	14	29.8	55.8	235.9	0.56	1.9	796.6	725.9	18.6	7
15-Jan-18	546	14	6	0.2	98.7	229	1.11	3	874.7	622.3	15.5	4.7
18-Jan-18	408	10	4	3	91.1	178.5	0.99	2.9	645.2	573.6	14	2.2
21-Jan-18	1062	26	6	11.4	71.4	258.7	0.77	3.5	2096.7	1435.7	15.5	9
23-Jan-18	408	2	2	4.8	35.5	156.9	0.31	2.8	161.3	230.7	11.5	0.4
31-Jan-18	306	5.8	8	7.6	72.3	265.4	0.79	2.3	823.8	571.3	16.6	5.3
1-Feb-18	174	4.2	6	12.4	45.71	91.4	0.39	1.9	99.1	196.5	15.5	1.4
Mean	918.2	16.3	10.3	10.7	53.9	215.3	0.55	2.99	1474.2	1066.7	16.5	12.5
Std dev.	944.6	14.2	8.3	9.8	28.1	86.9	0.35	1.08	1970.2	1198.4	2.9	18.9

## 4 Results

### 4.1 Relationships between variables

**Fig. 4:**
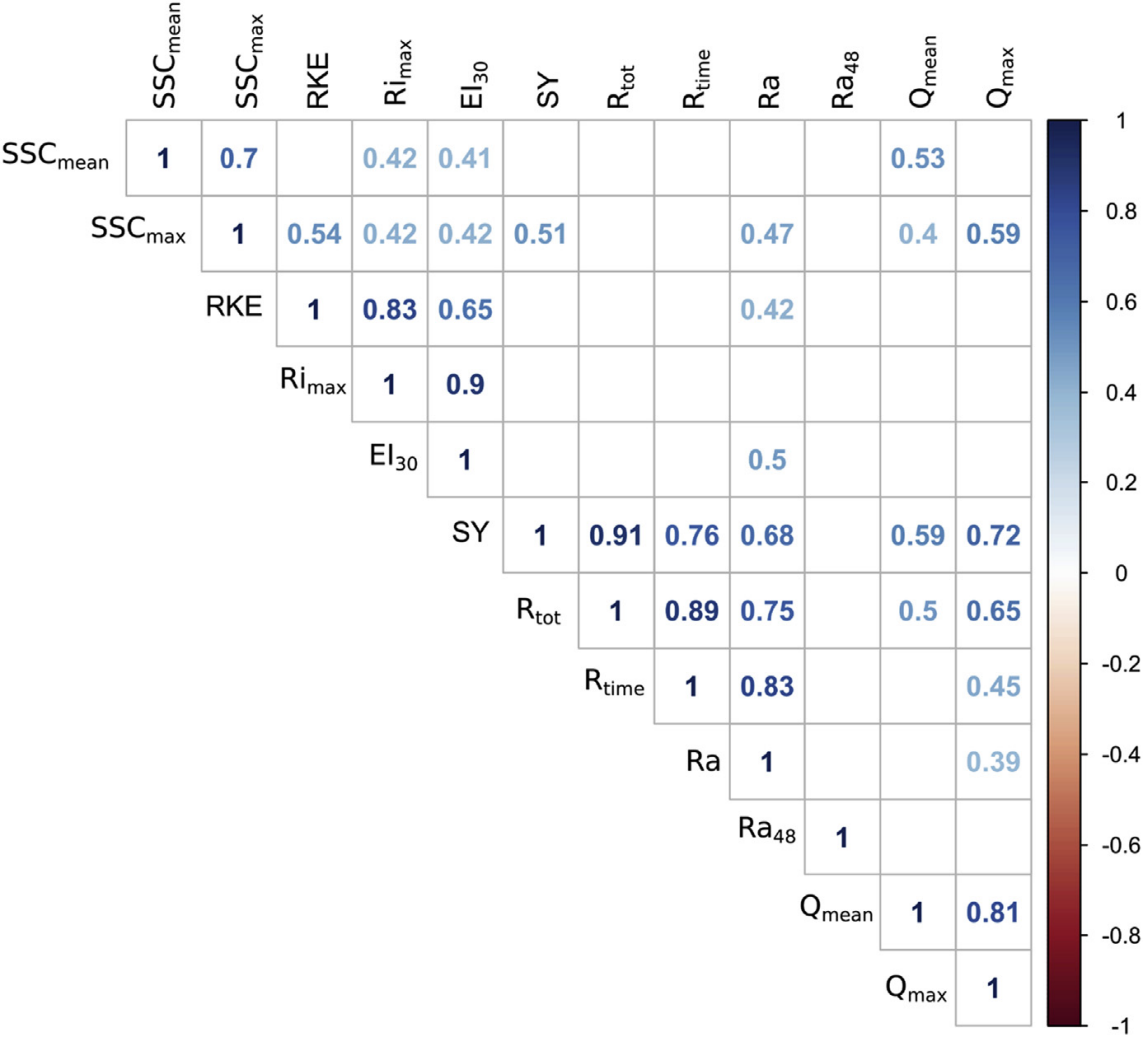
Pearson correlation matrix between all variables (n = 48 events). Coefficient r is considered significant at p = 0.01.

To identify the factors that may explain the measured hydro-sedimentological response at the outlet of the Pommeroye catchment, a Pearson correlation matrix was generated from all collected parameters. The results show significant correlations between the rainfall variables and the hydro-sedimentary response of the catchment area (Fig. 4). The highest relationship was found between the sediment yield (SY) and the total runoff (R_tot_; r = 0.91). Strong correlations were observed between the duration time of the event (R_time_), rainfall amount (Ra), and sediment yield (r = 0.76; r = 0.68). Reasonable correlations exist between the discharge variables (Q_mean_, Q_max_), sediment yield (SY) and total runoff (R_tot_) (r = 0.72; r = 0.65; r = 0.59; r = 0.5, respectively). A relationship between rainfall kinetic energy (RKE), rainfall erosivity index (EI_30_) and the maximum suspended sediment concentration (SSC_max_) is also observed (r = 0.54; r = 0.42).

**Fig. 5:**
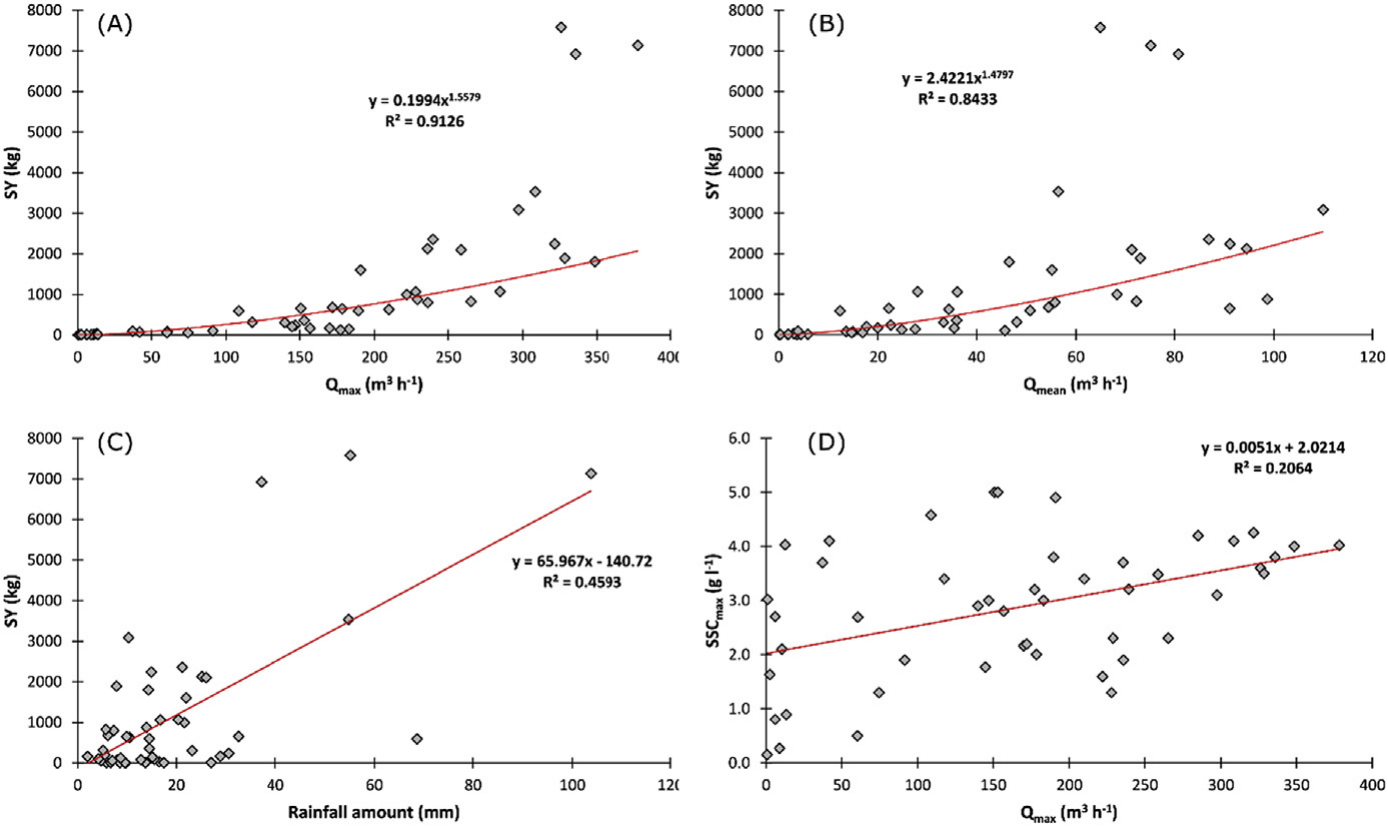
Relationship between (A) sediment yield (kg) and the maximum flow discharge (m^3^ h^−1^), (B) sediment yield (kg) and the mean flow discharge (m^3^ h^−1^), (C) sediment yield (kg) and the rainfall amount (mm), and (D) the maximum suspended sediment concentration (g L^−1^) and the maximum flow discharge (m^3^ h^−1^) after two years of monitoring in the Pommeroye catchment.

Excellent correlations were found between sediment yield and the maximum and mean flow discharge (r^2^ = 0.91 and r^2^ = 0.84, respectively; Fig. 5A and B) even with a dispersion for the biggest runoff events. The relationship between sediment yield and rainfall amount is statistically significant (r^2^ = 0.46), although it shows a wide scatter in the data (Fig. 5C). The maximum sediment yield at the outlet is observed when the rainfall amount exceeds 36 mm, except for one event occurring in summer 2016. Below the threshold of 36 mm, rainfall amount results in a more variable sediment yield at the outlet. For example, a rainfall amount of 15 mm results in a sediment yield varying between 50 and 2300 kg. The relationship between the peak flow and the maximum suspended sediment concentration was not statistically significant (r^2^ = 0.20). For a 4 g L^-1^ suspended sediment concentration the resulting peak flow varied between 10 and 380 m^3^ h^-1^ suggesting that the relationships between these two variables is more complex (Fig. 5D). The 48 h-antecedent rainfall does not show significant statistical relationships with the hydro-sedimentary response.

### 4.2 Sediment production variability

#### 4.2.1 First year of monitoring

**Fig. 6:**
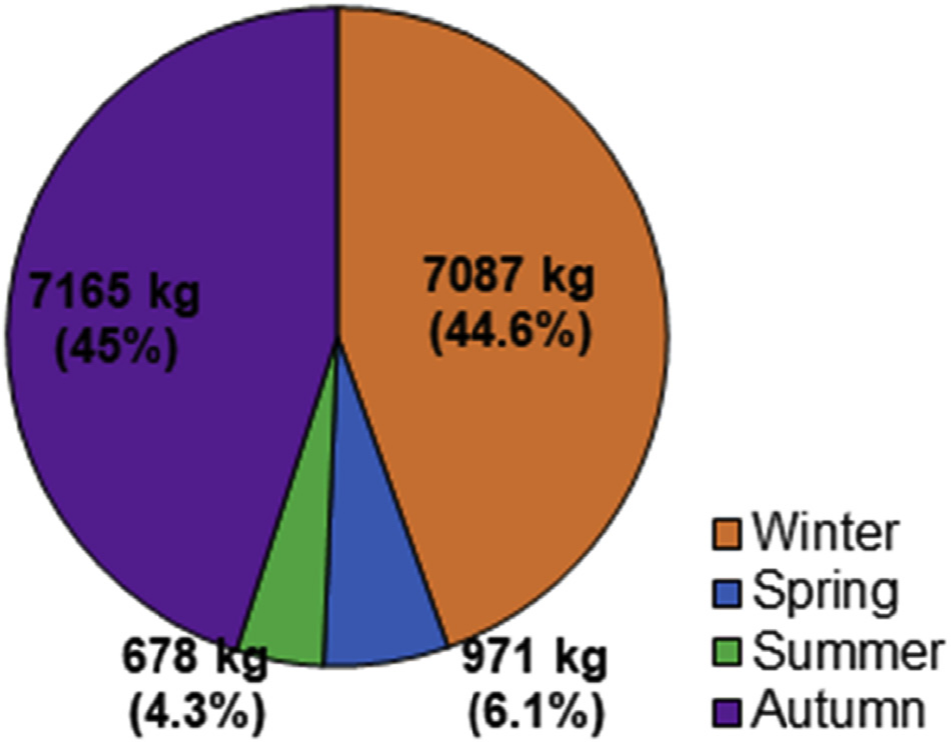
Seasonal distribution of the sediment export (%) at the outlet of the Pommeroye catchment for the first year of monitoring (April 2016–April 2017).

**Fig. 7:**
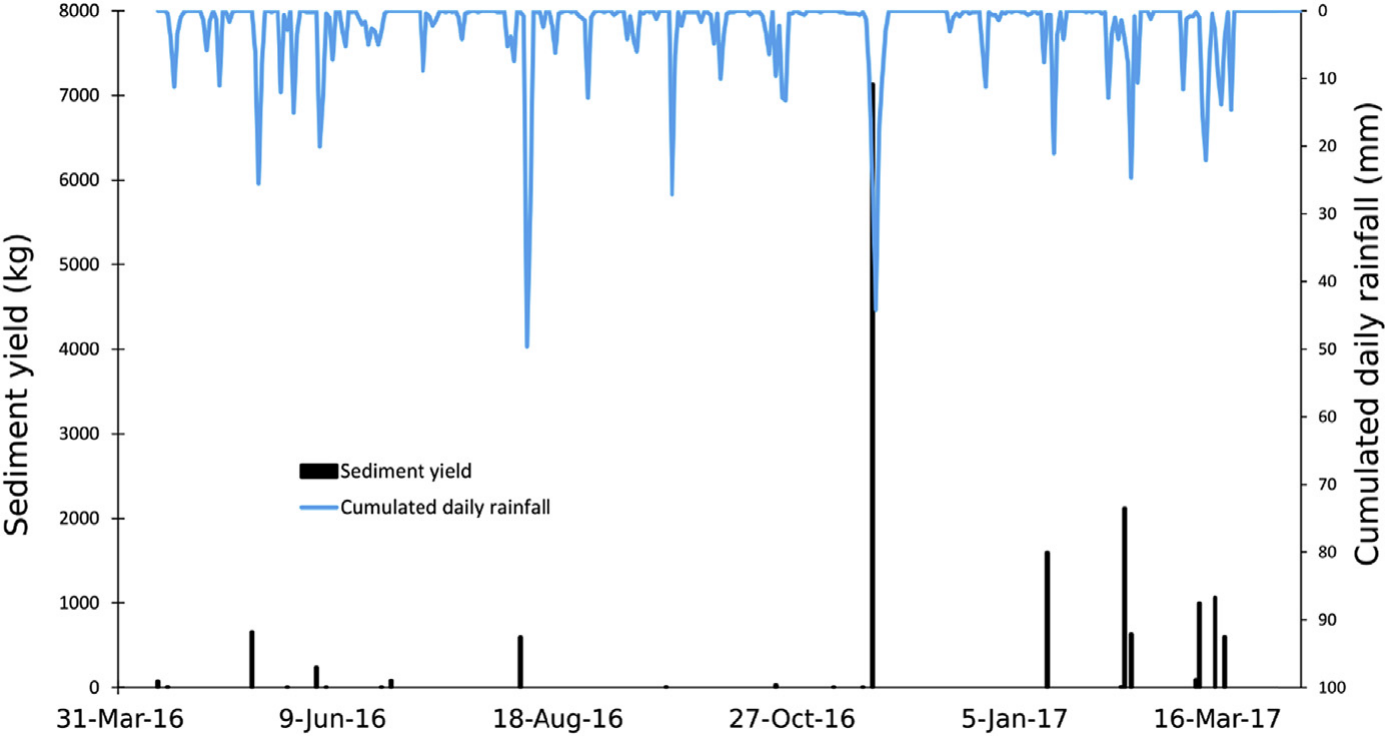
Sediment yield (kg) and cumulative daily rainfall (mm) recorded at the outlet of the Pommeroye catchment during the first year of monitoring (April 2016–April 2017).

From April 2016 to April 2017, the total SY recorded at the monitoring station reached 15901 kg (Fig. 6 and Fig. 7). The cumulated rainfall reached 827.1 mm, which is relatively low for the area (mean annual cumulative rainfall = 1000 ± 150 mm yr^-1^). Normalized to the catchment area (0.54 km^2^), the specific sediment yield (SSY) reached 29.4 t km^-2^ yr^−1^. The sediment yield shows a high heterogeneity between the different seasons. The seasonal SY, compared to annual SY in % is: (i) 971 kg in spring (6.1%), (ii) 678 kg in summer (4.3%), (iii) 7165 kg in autumn (45%), and (iv) 7087 kg in winter (44.6%).

In spring 2016, seven events contributed to the SY. Over this period, the total rainfall was 191 mm. The event of May 11, 2016 had the highest impact with a SY of 658 kg following a cumulative rainfall of 32.6 mm. This event contributed to 68% of the seasonal SY for spring. This may be explained by the fact that high rainfall intensities were measured, reaching up to 76 mm h^-1^ and high value for the RKE (24.8 J m^-2^ mm^−1^). For this event, maximum SSC was observed with a value of 5 g L^-1^.

In summer 2016, three events were recorded. Over this period, the total rainfall was 201.2 mm. On August 02, 2016 a SY of 591 kg and high cumulative rainfall (68.6 mm) was observed. This event contributed to 87% of the seasonal SY for summer 2016. Unlike during the spring season, rainfall intensities observed in summer were much lower. They reached 21 mm h^-1^ for the event on August 02, 2016. This explains why the observed SY is different between these two seasons.

In autumn 2016, four events were recorded including a peak event. Over this period, the total rainfall was 195.9 mm. The event of November 17, 2016 was due to a cumulative rainfall of 103.8 mm over five days. The SY for this event reached 7132 kg, equivalent to 45% of the total SY for the entire year. The maximum rainfall intensity was relatively high (18 mm h^-1^) but lower than in the previous seasons.

Winter 2016/2017 was the season with the highest frequency of rainfall events. A total of eight events were reported. Over this period, the total rainfall was 239 mm. The events of January 15, 2017 and February 02, 2017 contributed to 3717 kg (52.5%) of the total SY for winter season. These two events are the result of cumulative rainfalls of 22 and 25.2 mm, respectively. Four other events contributed to 3273 kg (46%) of the total SY in winter season. These events correspond to cumulative rainfall that ranged from 10.6 to 21.6 mm. During this period, lower heterogeneity between the events was observed, due to an almost equivalent cumulative rainfall for the different events and rainfall intensities that are generally low (between 4 to 12 mm h^-1^).

Over the entire first year of monitoring, it is noteworthy that during spring period, the highest sediment concentration was observed at the outlet. The autumn and winter seasons contributed equally to the total SY measured but strong heterogeneities between rainfall events was observed.

#### 4.2.2 Second year of monitoring

**Fig. 8:**
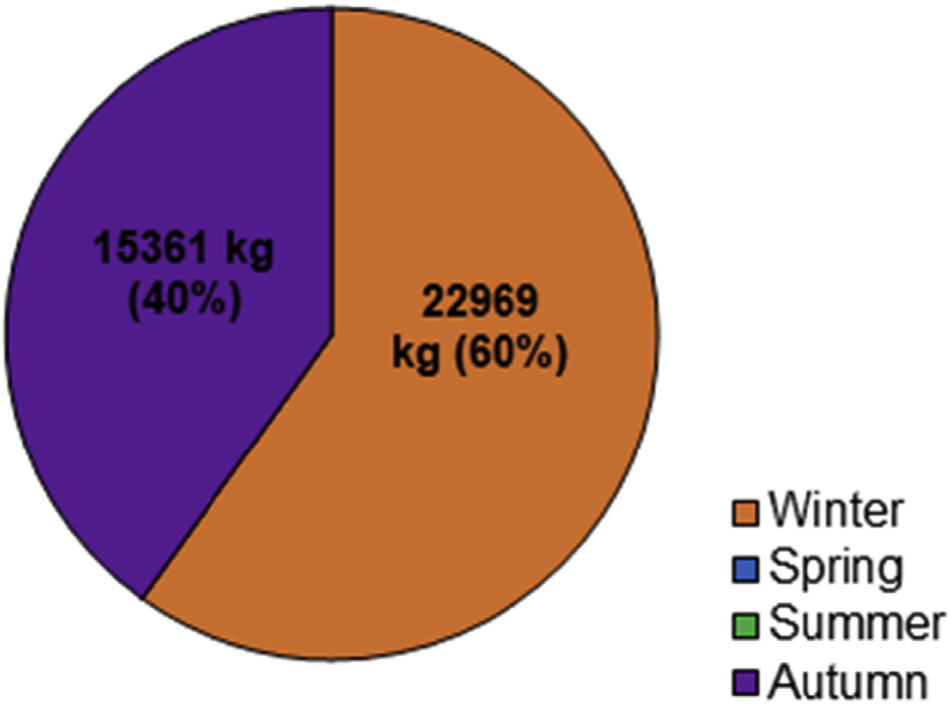
Seasonal distribution of the sediment export (%) at the outlet of the Pommeroye catchment for the second year of monitoring (April 2017–April 2018).

**Fig. 9:**
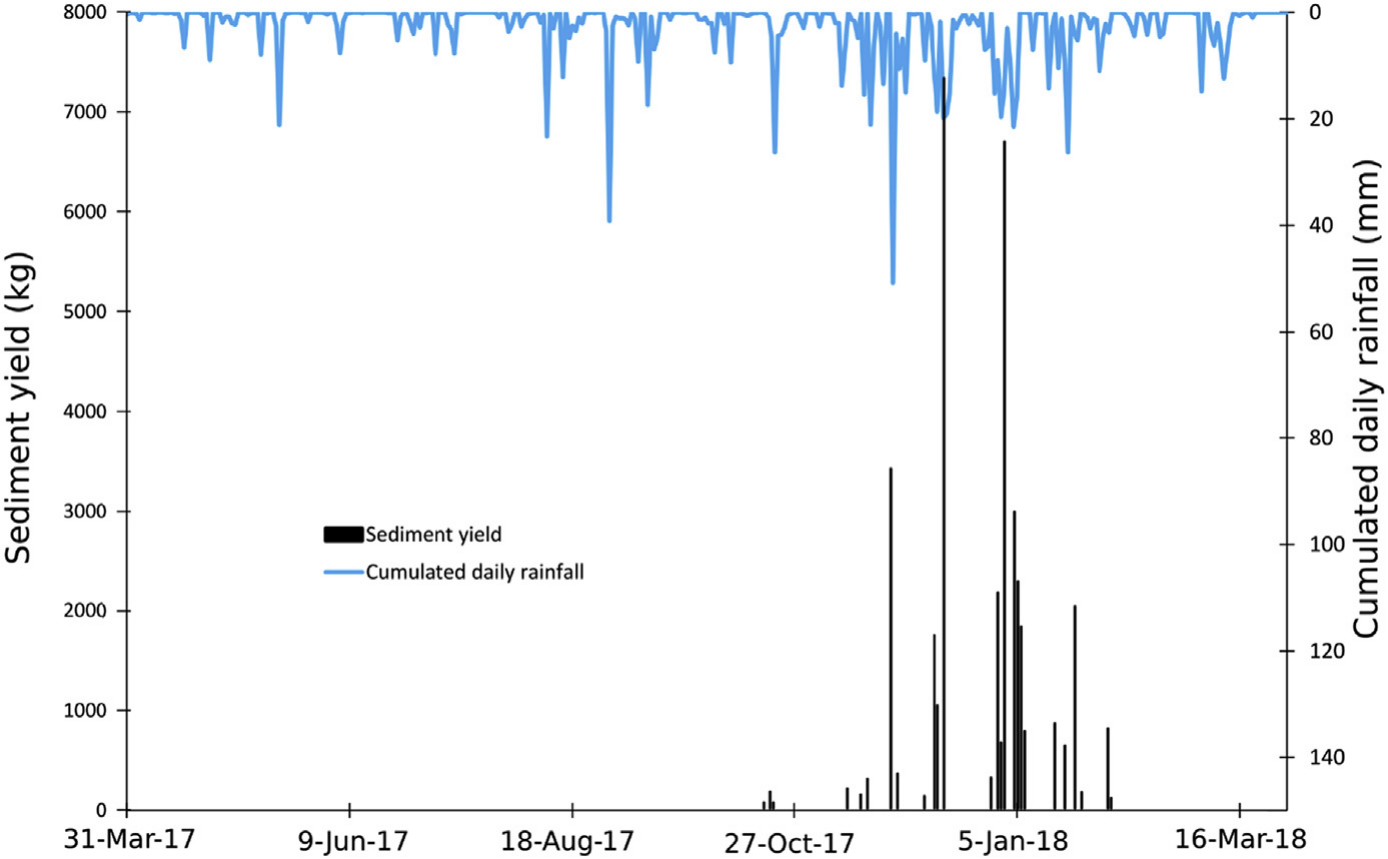
Sediment yield (kg) and cumulative daily rainfall (mm) recorded at the outlet of the Pommeroye catchment during the second year of monitoring (April 2017–April 2018).

From April 2017 to April 2018, the total SY recorded at the monitoring station reached 38330 kg (Fig. 8 and Fig. 9). The cumulated rainfall reached 965.8 mm which is higher than previous year. Normalized by the catchment area (0.54 km^2^), the SSY reached 70 t km^-2^ yr^−1^. The seasonal SY, compared to annual SY in % is: (i) 15361 kg in autumn (40%) and (ii) 22969 kg in winter (60%). The two other seasons are not represented here as no events were recorded at the monitoring station.

In autumn 2017, twelve events were recorded for a total rainfall of 367.5 mm, thus 171.6 mm more than during the previous year for the same season. Two events contributed to 72% of the seasonal SY for autumn 2017. For the event of November 27, 2017, the measured SY can be explained by a high rainfall amount (54.8 mm) and the highest maximum rainfall intensity recorded for this season (24 mm h^-1^). For the event on December 13, 2017, the rainfall amount was similar (55.2 mm) but the maximum rainfall intensity was lowest (8 mm h^-1^). The observed SY can be explained by a long duration of the rainfall event (3 days) and a high amount of 48 h-antecedent rainfall (20.6 mm).

In winter 2017/2018, fourteen events were recorded for a total rainfall of 323.2 mm, thus 84.2 mm more than in the previous year for the same season. 74% of the seasonal SY was produced between December 29, 2017 and January 5, 2018. The main event of December 31, 2017 which contributed to 30% showed the longest duration of rainfall event (2694 min). The rainfall amount was 37.2 mm with a high maximum rainfall intensity (30 mm h^-1^) and a high amount of 48 h-antecedent rainfall (21.4 mm). The events following this main event were of relatively short rainfall event duration between 192 and 420 min. The rainfall amount was variable and comprised values between 5.2 and 21.2 mm, just as the maximum rainfall intensity, which varied between 4 and 22 mm h^-1^. Five events were characterized by a high amount of 48 h-antecedent rainfall varying between 13.6 and 29.8 mm. After January 15, 2018, six more events were recorded. They contributed to 20% to the seasonal SY with a rainfall amount ranges from 2 to 26 mm and durations between 174 and 1062 min. Rainfall intensity were relatively low from 2 to 8 mm h^-1^ and the 48 h-antecedent rainfall ranged from 0.2 to 12.4 mm.

### 4.3 Seasonal variability

**Fig. 10:**
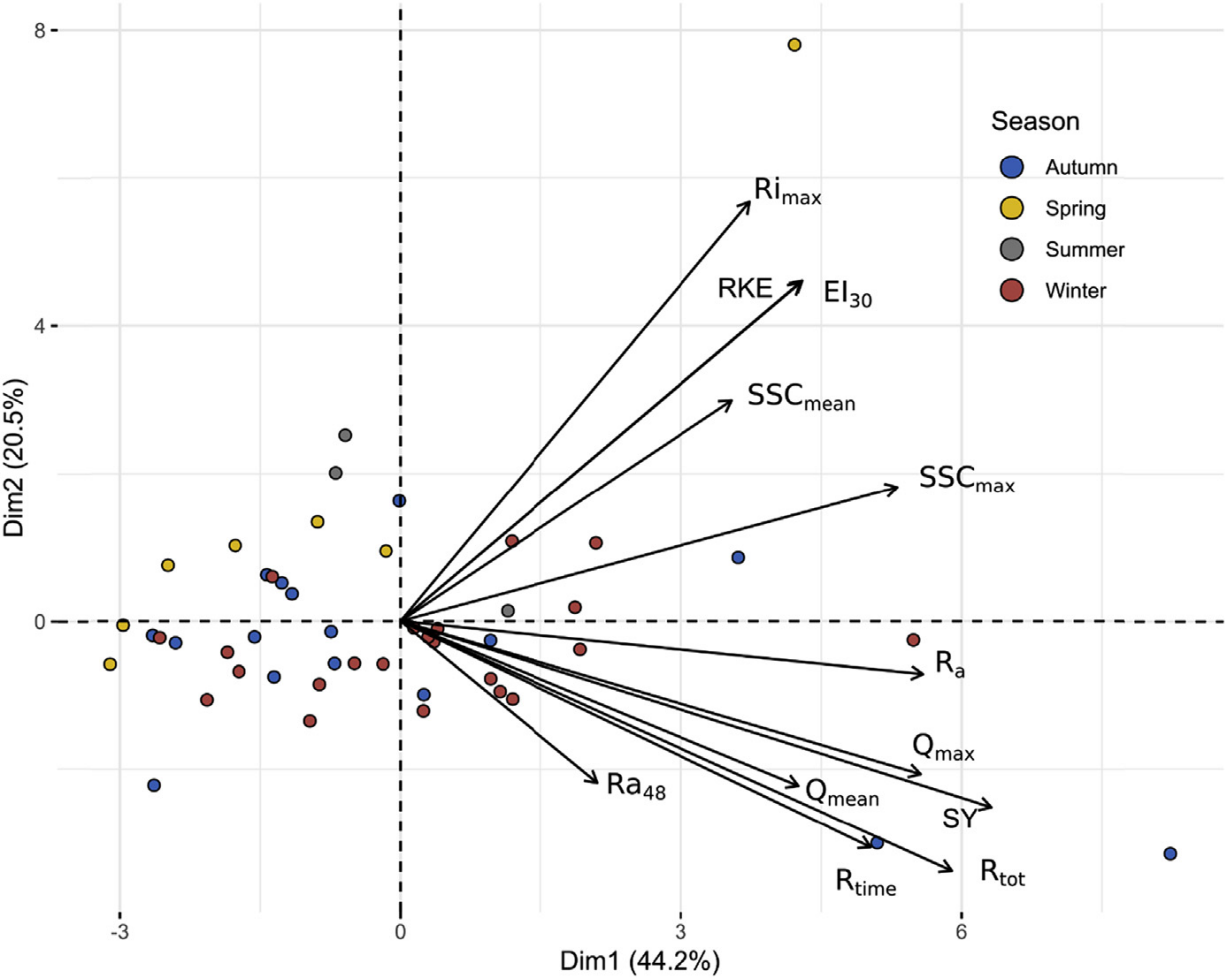
Bi-plot of PCA results on dimensions 1–2 for the 48 events and their hydro-sedimentary parameters: (i) duration of the rainfall event (R_time_, min), (ii) rainfall amount (Ra, mm), (iii) max 6 min rainfall intensity (Ri_max_, mm h^−1^), (iv) 48 h-antecedent rainfall (Ra_48_, mm), (v) mean flow and peak flow (Q_mean_, Q_max_, m^3^ h^−1^), (vi) mean and maximum SSC concentration (SSC_mean_, SSC_max_, g L^−1^), (viii) sediment yield (SY, kg), (ix) total runoff (R_tot_, m^3^), (x) rainfall kinetic energy (RKE, J m^−2^ mm^−1^), and (xi) rainfall erosivity index (EI_30_, MJ mm ha^−1^ h^−1^).

**Fig. 11:**
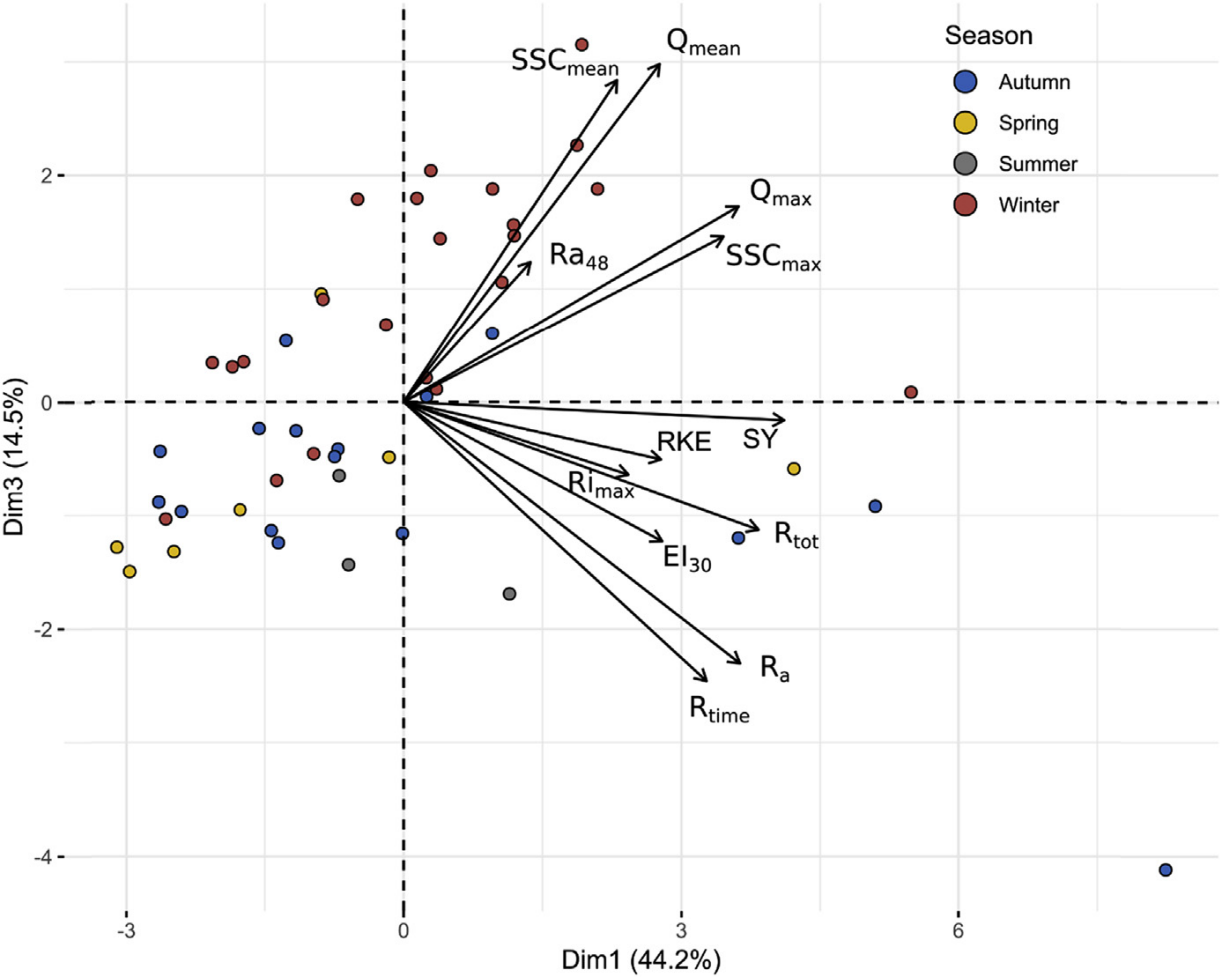
Bi-plot of PCA results on dimensions 1–3 for the 48 events and their hydro-sedimentary parameters: (i) duration of the rainfall event (R_time_, min), (ii) rainfall amount (Ra, mm), (iii) max 6-min rainfall intensity (Ri_max_, mm h^−1^), (iv) 48 h-antecedent rainfall (Ra_48_, mm), (v) mean flow and peak flow (Q_mean_, Q_max_, m^3^ h^−1^), (vi) mean and maximum SSC concentration (SSC_mean_, SSC_max_, g L^−1^), (viii) sediment yield (SY, kg), (ix) total runoff (R_tot_, m^3^), (x) rainfall kinetic energy (RKE, J m^−2^ mm^−1^), and (xi) rainfall erosivity index (EI_30_, MJ mm ha^−1^ h^−1^).

The eigen-values (Table 4) provide the percentage of the explained variance and the cumulative variance of the principal dimensions. The first three dimensions explain 79.2% of the total variance: dimension 1 accounts for 44.2%, dimension 2 for 20.5%, and dimension 3 for 14.5%. The square cosines of variables indicate the best-described variables on each principal dimension. Dimension 1 is correlated to R_time_ (0.48), Ra (0.59), Q_max_ (0.59), SSC_max_ (0.54), SY (0.76), and R_tot_ (0.66). Dimension 2 is correlated to Ri_max_ (0.61), RKE (0.40), and EI_30_ (0.40). Dimension 3 is correlated to Q_mean_ (0.40) and SSC_mean_ (0.36). Ra_48_ is correlated to Dimension 4 (0.72) which represented only 7.2% of the total variance. The bi-plot graphs (Fig. 10 and Fig. 11) allow visualizing the information on both, individual samples and variables.

**Table 4:** Eigen-values, percentages of variance explained, and cumulative variance of principal dimensions for the principal component analysis.

	Eigen-value	Percentage of explained variance (%)	Cumulative percentage of explained variance (%)
Dim 1	5.3	44.2	44.2
Dim 2	2.46	20.5	64.7
Dim 3	1.75	14.5	79.2
Dim 4	0.87	7.2	86.4
Dim 5	0.57	4.8	91.2
Dim 6	0.45	3.8	95
Dim 7	0.24	2	97
Dim 8	0.19	1.5	98.5
Dim 9	0.08	0.7	99.2
Dim 10	0.04	0.4	99.6
Dim 11	0.03	0.2	99.8
Dim 12	0.02	0.2	100

In the first space Dim1-Dim2, we observe a classification between the different events (Fig. 10). On Dim1^+^/Dim2^+^, a minority of events are represented and are those characterized with the highest values of the following parameters: Ri_max_, RKE, EI_30_, SSC_mean_, SSC_max_. These events are the most erosive and occurred in the four seasons. The event of May 11, 2016 seems to have a high influence on the projection of the principle component analysis (PCA) due to a high value of climatic parameters. This event was the most erosive with very high rainfall intensities (76 mm h^-1^) and a strong erosivity power (RKE = 24.8 J m^-2^ mm^−1^; EI_30_ = 236.2 MJ mm ha^−1^ h^−1^), which results on a high suspended sediment concentration at the outlet of the catchment. Nevertheless, these events are not characterized by the highest sediment yield at the outlet. Events with the highest values for flow discharge and sediment yield are represented in Dim1^+^/Dim2^-^ and occurred mostly in autumn and winter. These events are characterized by the highest values of sediment yield induced by highest values of rainfall characteristics (Ra, R_tot_, R_time_). In the two other spaces (Dim1^-^/Dim2^+^; Dim1^-^/Dim2^-^), the events occurred mainly in winter, spring, and autumn. As there is no discriminating parameter on this side, these events are characterized by the lowest values of the different parameters previously cited.

In the second space Dim1/Dim3, the classification of the events shows some additional information (Fig. 11). A major part of the winter events is represented in the Dim1^+^/Dim3^+^ space and is characterized by high values of Q_mean_, Q_max_, SSC_mean_, and SSC_max_. In this space, the parameter Ra_48_ appears to be discriminating. In Dim1^+^/Dim3^-^, few events of the season autumn, spring, and summer are represented. They are characterized by the highest values of SY, EI_30_, Ri_max_, Ra, RKE, and R_time_. However, most of the events occurring in these three seasons are represented in the space Dim1^-^/Dim3^-^ and are largely influenced by the value of the previously cited parameters.

## 5 Discussion

A high intra-annual variability of the hydro-sedimentary response is observed at the outlet of the Pommeroye catchment. The experimental station measured a specific sediment yield between 29.4 and 70 t km^-2^ yr^−1^ over two years of monitoring while the intra-annual variability of cumulative rainfall is low with a difference of 138.7 mm. The high variability of the hydro-sedimentary response is explained by the 48 erosive events recorded which show a strong heterogeneity. Sediment flux for a single event ranges from 0.6 to 7131.8 kg, with an average of 722.8 kg. The total runoff between discrete events also shows strong variations ranging from 0.57 to 8630.5 m^3^. This variability of the hydro-sedimentary response can be mainly explained by different types of rainfall events. Cumulative rainfall for a single event shows a high variability (i.e. from 2 to 103.8 mm) as well as the recorded maximum rainfall intensities (i.e. from 2 to 76 mm h^-1^).

The amount of exported sediment is consistent with values observed in similar studies areas. The specific sediment yield is higher than those observed by Grangeon et al. (2017) across the main Louroux subcatchments (France) where sediment fluxes varied from 1 to 38 t km^-2^ yr^−1^. Our results on SY are also higher than those published by Lefrançois (2007) for the French catchments of the Moulinet and the Violettes (i.e. 25.4 and 36 t km^-2^ yr^−1^; catchment area = 4.53 and 2.24 km^2^, respectively) and those observed by Laignel et al. (2006) and Landemaine (2016) in larger catchments in Normandy, France (Austreberthe: 16 t km^-2^ yr^−1^ and Andelle: 21 t km^-2^ yr^−1^). On the other hand, the SY results for the two years monitoring of the Pommeroye catchment are lower than those measured by Walling et al. (2002) in UK catchments (Belmont, Jubilee, and Lower Smisby; catchment area = 1.5, 0.3, and 2.6 km^2^, respectively) with a specific sediment yield between 70.6 and 181.1 t km^-2^ yr^−1^.

A strong variability is observed between the two years of monitoring at the Pommeroye catchment with a difference of 40.6 t km^-2^ yr^−1^. Supplemental years of monitoring on the Pommeroye catchment would be useful to increase the representativeness of the measured sediment yield. Nevertheless, similar observations were made by Walling et al. (2002) for the catchment of Jubilee and New Cliftonthorpe (NC) in UK where the specific sediment yield varied from 81.1 to 181.1 t km^-2^ yr^−1^ over two years of monitoring for the Jubilee, and from 0.6 to 122.4 t km^-2^ yr^−1^ for the NC. Comparable approaches in Belgium have also shown that hydro-sedimentary responses were highly variable between two distinct hydrologic years (Vandaele and Poesen, 1995; Poesen et al., 1996). Walling et al. (2002) also point out that this variability is not only due to the variability of rainfall amount but also to the temporal variability of the rainfall during the year. This is particularly true in the Pommeroye catchment where rainy events were spread out in time during the first year whereas they mainly grouped over a shorter period during the second year. This succession of rainy events during a short time interval may cause a saturation of the infiltration capacity of the soil and the creation of a slaking crust in loess environments (Le Bissonnais et al., 2002).

Because of the heterogeneity of the rainfall events, only 6% of the erosive events exported 21 t of sediment, i.e. 40% of the sediment flux transported over the two years. Some authors also observed that a few numbers of erosive episodes were responsible for a large part of the exported sediment. Indeed, Nu-Fang et al. (2011) showed that 90% of the hydro-sedimentary flux could be produced by only nine erosive events for a hydrological year in the Wangjiaqiao catchment near the Three Gorges dam in China. Estrany et al. (2009) also observed for the Mediterranean catchment that 90% of the hydro-sedimentary flux was transported during only 0.13% of the time over the studied period. Similar observations were made by Lana-Renault and Regüés (2009) in Spain where 75% of the hydro-sedimentary flux were produced by only 15% of the erosive events over the studied period. These results clearly underline the variability of the hydro-sedimentary production in small agricultural catchments for which most of the hydro-sedimentary fluxes are produced by a small number of events. Strong seasonal variability was identified over the two years of monitoring at the Pommeroye catchment. Most of the hydro-sedimentary transfer was produced in winter (55%) and autumn (42%). Only 3% of the sediment flux is produced during the spring and summer periods although the rainfall intensities are the most important. This can be mainly explained by the state of the soil surface, more particularly by a better crop cover on the agricultural plots which plays an important role of protection against the rainfall impact.

The employed statistical analyses showed that SSY and Q_max_ have the best correlation (r^2^ = 0.91). It is reasonable to expect a high degree of correlation between these two variables considering that runoff is produced by rainfall excess in Hortonian environments, and where Q_max_ is a function of rainfall intensity and duration. It has been noticed in the literature that Q_max_ is a meaningful variable because it exerts influence on both the production and the transfer functions of sediment dynamics (Duvert et al., 2012). Positives correlations between sediment and rainfall variables were also observed (SY vs. R_time_, and SY vs. R_a_), but not as robust as the relationships between SSY and discharge variables. The correlation between the amount of rainfall and the sediment yield shows a threshold at 36 mm for which the erosive events are the most important. Below this threshold, a dispersion of the data cloud is observed, which seems to indicate that other forcing parameters must be considered, such as the surface state of the soils. As emphasized by Evrard et al. (2010), the state of the soil surface result from rainfall conditions and the vegetation growing system in place. In addition, agricultural operations modify the state of the soil surface and are practiced at different times of the year. Depending on the temporal variability of soil surface conditions (soil erodibility, roughness, crop cover) in the catchment, it can be assumed that the erosion processes will show significant spatial and temporal variability throughout the year (Cerdan et al., 2002b, Cerdan et al., 2002a). To better assess the impact of these parameters on the hydro-sedimentary response, monitoring of the soil surface conditions over time, with an adapted spatio-temporal resolution, could give further valuable information. The consideration of cultural practices and soil quality is therefore a continuation of this work, which is presented in further detail in Patault (2018).

Surprisingly, the amount of 48 h-antecedent rainfall is not correlated with the hydro-sedimentary response. Thus, the parameter cannot be considered as an explanatory factor even though this parameter is commonly used in runoff erosion models developed for catchments located on the European loess belt: i.e. *STREAM* and *WATERSED* models (Cerdan et al., 2002b, Cerdan et al., 2002a; Souchère et al., 2003; Landemaine, 2016). In addition, maximum rainfall intensities are not correlated with the hydro-sedimentary response (expect for the suspended sediment concentration). These results are contradictory with those published by Nu-Fang et al. (2011) and suggest that the modification of farming practices and the farmer's efforts to get an important crop cover over the plots throughout the year allows a reduction in the impact of the rain splash effect. In addition, the rainfall kinetic energy seems to be a more representative parameter of detachment and transport of suspended particulate matter than the rainfall intensity in our study site and shows a reasonably good correlation with the maximum suspended sediment concentration observed at the outlet of the Pommeroye catchment.

The correlation between the maximum sediment concentration and the peak flow was found as not statistically significant, indicating that the relationship is more complex between these two variables. As pointed out by Williams (1989), hysteresis effects can be observed during erosive episodes and multiple sediment sources can be mobilized. Sources that cause sediment remobilization may include diffuse erosion on plots or concentrated erosion through ephemeral gullies. In the Pommeroye catchment, the hydro-sedimentary response is complex, and it is difficult to identify the exact sediment sources responsible of the sediment transfer via the flood hydrographs. An alternative approach could be to quantify the contribution of these different sources via the use of a GIS model. These approaches have been successfully used by Cerdan et al., 2002b, Cerdan et al., 2002a and Stolte et al. (2003). For example, the *STREAM* model (Cerdan et al., 2002a; Souchère et al., 2003) and more recently the *WATERSED* model (Landemaine, 2016) allow the prediction of diffuse and concentrated erosion in catchments. These models considered the catchment morphology, the rainfall characteristics, and the soil surface conditions to quantify the hydro-sedimentary response for a specific rain event and to evaluate the spatial variability of sediment remobilization. We consider this future particularly suitable for the Pommeroye catchment, intending to allow the identification of the spatial variability of the sediment sources during an erosive episode and to quantify their respective contributions.

These two years of experimental monitoring are a first step for an on-going erosion reduction project (Patault, 2018) and will be used to calibrate the *WATERSED* erosion model. Moreover, this will allow to evaluate the efficiency of hypothetical erosion control measures (fascines, hedges, grass strips) on the studied catchment. The observations on the Pommeroye catchment and the *WATERSED* modelling also should allow to upscale to larger areas (Canche watershed) which have the same erosion problems (e.g. Pataut et al., 2019) but not the same logistics in terms of monitoring.

## 6 Conclusion

This study reports on a detailed record of the hydro-sedimentary response to rainfall events in the small agricultural catchment of the Pommeroye in Northern France. The research was based on a high-frequency (6 min) monitoring of rainfall, runoff, and suspended sediment transport over a period of two hydrological years (April 2016–April 2018), in a challenging context where the studied catchment is a lacking a perennial hydrographic network. A high inter-annual variability of the sediment yield has been observed. Over two years, the specific sediment yield ranges from 29.4 to 70 t km^-2^ yr^−1^ suggesting a large heterogeneity of the erosive events. Most of the sediment was transported in winter (55%) and autumn (42%) whereas it was less significant in summer and spring (3%). At the event scale, the results showed high variability in their hydro-sedimentary response. A small number of events (6%) were responsible for a large proportion of the sediment yield (40%). The multivariate statistical analyses showed that the best correlation is observed between SSY and Q_max_ and that the rainfall variables: R_a_ and R_time_, are the most relevant factors controlling the hydro-sedimentary response. Rainfall intensity and rainfall kinetic energy explained the highest values of suspended sediment at the outlet, while the 48 h-antecedent rainfall is not statistically significant with the hydro-sedimentary parameters. These results confirm the high variability of the hydro-sedimentary response to rainfall events, the complexity of the erosion processes in the agricultural plains of North-Western Europe, and improve our understanding of the possible connections or disconnections between the water and the sediment transport pathways. The findings are important for watershed stakeholders, as it enhances our understanding on the spatio-temporal variability of sediment fluxes induce by mudflows and their main controlling factors.

## Declarations

### Author contribution statement

Edouard Patault: Conceived and designed the experiments; Performed the experiments; Analyzed and interpreted the data; Contributed reagents, materials, analysis tools or data; Wrote the paper.

Claire Alary, Christine Franke: Conceived and designed the experiments; Analyzed and interpreted the data; Contributed reagents, materials, analysis tools or data; Wrote the paper.

Arnaud Gauthier, Nor-Edine Abriak: Analyzed and interpreted the data; Contributed reagents, materials, analysis tools or data; Wrote the paper.

### Funding statement

This work was supported by the Mines-Telecom Institute of Lille-Douai, with additional funding provided by the Water Agency of Arthois-Picardie (QUASPER project).

### Competing interest statement

The authors declare no conflict of interest.

### Additional information

No additional information is available for this paper.

## Footnotes

1 R Core Team (2008). R: A Language and Environment for Statistical Computing. R Foundation for Statistical Computing, Vienna, Austria. URL https://www.R-project.org/.

2 https://cran.r-project.org/web/packages/FactoMineR/index.html.

3 https://cran.r-project.org/web/packages/corrplot/index.html.
